# Dietary Broccoli Alters Rat Cecal Microbiota to Improve Glucoraphanin Hydrolysis to Bioactive Isothiocyanates

**DOI:** 10.3390/nu9030262

**Published:** 2017-03-10

**Authors:** Xiaoji Liu, Yanling Wang, Jennifer L. Hoeflinger, Bárbara P. Neme, Elizabeth H. Jeffery, Michael J. Miller

**Affiliations:** 1 Department of Food Science and Human Nutrition, University of Illinois, 905 S. Goodwin Ave., Urbana, 61801 IL, USA; xiaoji2@illinois.edu (X.L.); ywang436@illinois.edu (Y.W.); jlwinric@illinois.edu (J.L.H.); ejeffery@illinois.edu (E.H.J.); 2 Department of Food Technology, Federal Rural University of Rio de Janeiro, 23.897-000 Seropédica, Brazil; barbara.neme@hotmail.com

**Keywords:** broccoli, glucoraphanin, isothiocyanate, rat, gut microbiome

## Abstract

Broccoli consumption brings many health benefits, including reducing the risk of cancer and inflammatory diseases. The objectives of this study were to identify global alterations in the cecal microbiota composition using 16S rRNA sequencing analysis and glucoraphanin (GRP) hydrolysis to isothiocyanates ex vivo by the cecal microbiota, following different broccoli diets. Rats were randomized to consume AIN93G (control) or different broccoli diets; AIN93G plus cooked broccoli, a GRP-rich powder, raw broccoli, or myrosinase-treated cooked broccoli. Feeding raw or cooked broccoli for four days or longer both changed the cecal microbiota composition and caused a greater production of isothiocyanates ex vivo. A more than two-fold increase in NAD(P)H: quinone oxidoreductase 1 activity of the host colon mucosa after feeding cooked broccoli for seven days confirmed the positive health benefits. Further studies revealed that dietary GRP was specifically responsible for the increased microbial GRP hydrolysis ex vivo, whereas changes in the cecal microbial communities were attributed to other broccoli components. Interestingly, a three-day withdrawal from a raw broccoli diet reversed the increased microbial GRP hydrolysis ex vivo. Findings suggest that enhanced conversion of GRP to bioactive isothiocyanates by the cecal microbiota requires four or more days of broccoli consumption and is reversible.

## 1. Introduction

Broccoli belongs to the glucosinolate (GSL)-containing family of brassica vegetables. The major GSL in broccoli is glucoraphanin (GRP) which, upon hydrolysis, produces the bioactive isothiocyanate (ITC) sulforaphane (SFN), shown to slow or prevent cancer in humans [1]. One major mechanism of action of SFN is upregulation of several phase II detoxification enzymes, including NAD(P)H: quinone oxidoreductase 1 (NQO1) [2]. Here we use increased NQO1 levels as a measure of broccoli-induced bioactivity.

The formation of SFN from GRP is catalyzed by the plant enzyme myrosinase, active during chewing or crushing when broccoli is consumed raw or lightly steamed [3]. When broccoli and other brassica vegetables are cooked more vigorously, the plant myrosinases are inactivated and, thus, are unable to hydrolyze GSLs [4]. Even though many US consumers prefer cooked broccoli, epidemiological studies suggest that frequent consumption of brassica vegetables decreases the risk for a number of cancers, including breast, colon, and prostate cancers [5]. This suggests that GSLs undergo hydrolysis to ITC following ingestion, in the absence of active plant myrosinase. Several microorganisms isolated from the mammalian gut, including lactic acid bacteria [6], *Enterobacteriaceae* [7], *Bifidobaicterium* spp. [8], and *Bacteroides* spp. [9], appear to have myrosinase-like glycoside hydrolases that cleave GSL. However, although research shows direct evidence for GRP hydrolysis ex vivo and in vivo to bioactive SFN by cecal microbiota, SFN is only found in trace amounts [10]. Comparatively inactive nitriles [11,12], not bioactive ITC, are typically reported as products of microbial GSL hydrolysis ex vivo, as reviewed in [3], and ingestion of cooked broccoli typically provides only about one tenth the amount of SFN as that from raw broccoli hydrolyzed by plant myrosinase during digestion [13,14,15]. These findings suggest that if hydrolysis to SFN by microbiota could be enhanced, consumers might obtain greater health benefits from cooked broccoli. Although microbial glycoside hydrolase has been shown to be involved in the degradation of GSL with the pathogenic *Escherichia coli* O157:H7 [16] and a soil *Citrobacter* spp. [17], it is not yet clear which gut commensal microorganism(s) are responsible for GSL conversion to ITC. In addition, why most ex vivo studies identify nitriles, rather than ITC, as the product of hydrolysis is also unknown. Furthermore, while there have been some studies that have evaluated the impact of broccoli on the gut microbiome [18,19], no correlation studies relating gut microbial community composition and GRP hydrolysis have been reported. Our hypothesis is that frequent consumption of broccoli will promote a change in the gut microbial community resulting in increased microbial SFN production, with increased NQO1 activity in the host tissues, such as the colon and liver.

In this study, the effect of feeding broccoli and GRP on the composition of rat cecal microbiota was analyzed using Illumina 16S rRNA sequencing. We also determined changes in GRP hydrolytic activity ex vivo by cecal microbiota and NQO1 activity in rat colonic mucosa and liver.

## 2. Materials and Methods 

### 2.1. Animals and Diets

Animal use for Project Protocol 15042 was approved on 20 March 2015 by the Illinois Institutional Animal Care and Use Committee according to National Institutes of Health guidelines. Fischer 344 rats weighing 120–140 g, were purchased from Harlan (Colony 217, Indianapolis, IN, USA; Colony 208, Frederick, MD, USA) and housed individually in cages with food and water ad libitum. Rats from two colonies were used to evaluate the impact on broccoli hydrolysis of reported differences in microbial composition among colonies [20]. Rats were acclimated to a powdered AIN93G diet (Table 1) for five days prior to transition to experimental diets. All diets were balanced to provide similar macronutrient content to the AIN93G diet (Table 1) according to the USDA National nutrient database [21].

Commercially available pre-cooked, frozen broccoli (cooked broccoli; CB) was freeze-dried before incorporation into the diets. The CB was confirmed free of both myrosinase activity and SFN. A raw broccoli (RB) diet was prepared by freeze-drying commercial fresh broccoli. Glucoraphanin-free broccoli was prepared by the addition of excess exogenous myrosinase (thioglucosidase from *Sinapis alba*, Sigma-Aldrich, Carlsbad, CA, USA) to cooked broccoli, then incubating for 8 h at room temperature to complete hydrolysis (hydrolyzed cooked broccoli; CB-H), followed by additional freeze drying; some SFN but no GRP remained. A GRP-rich powder was prepared from broccoli seed raffinate (SFN glucosinolate broccoli raffinate, CS Health, Louisville, KY, USA) as previously described [22], and was added to the GRP diet (0.41% *w*/*w*) to achieve the same GRP concentrations as that found in the CB diet (10% broccoli *w*/*w*). All rats were anaesthetized with ketamine/xylazine (87 and 13 mg/mL, respectively), blood drawn, and then killed by cervical dislocation. The ceca were ligated anteriorly and distally, surgically removed, and immediately transferred to an anaerobic chamber to remove the microbiome for DNA isolation and for GRP hydrolysis ex vivo.

### 2.2. Experimental Design

Rats from Colonies 208 and 217 were used in Study 1 and only rats from Colony 217 were used in Studies 2 and 3. Study 1: 18 rats from Colony 208 were acclimated to AIN93G (control; CON) diet, randomized to six groups of three and fed the CB diet ad libitum for 0, 1, 2, 4, 7, or 14 days; the study was then repeated with another 18 rats, but from colony 217. Study 2: To separate the effects of GRP and non-GRP components of broccoli, 32 rats from Colony 217 were randomized into four treatment groups (*n* = 8) and fed; (1) CON diet; (2) CB diet; (3) GRP diet; or (4) CB-H diet (containing no GRP), for four days. Study 3: To examine the effects of raw broccoli feeding and of withdrawal from broccoli, 9 rats (Colony 217) were divided into three groups of three and fed; (1) CON diet; (2) RB diet for four days; or (3) RB diet for four days then the CON diet for three days.

### 2.3. Cecal Microbiota Profiling by 16S rRNA Sequencing

Total DNA was extracted from 40 mg of cecal contents using QIAmp DNA stool Mini Kit (Qiagen, Alameda, CA, USA) with bead-beating [23,24]. Concentration and quality of DNA was measured using NanoDrop (Thermo Scientific, Franklin, MA, USA). The 16S rRNA sequencing was performed by the DNA Sequencing Group at the Roy J. Carver Biotechnology Center, University of Illinois. The V3-V5 variable regions of the 16S rDNA were amplified using primers (forward F357: 5′-CCTACGGGAGGCAGCAG-3′ and reverse R926: 5′-CCGTCAATTCMTTTRAGT-3′). The quality of the PCR products was analyzed using an Agilent 2100 BioAnalyzer.

Paired-end reads were generated with the Illumina^®^ MiSeq platform for each sample, at a read length of 250 nt. The reads were demultiplexed and quality-filtered using Trimmomatic [25] (version 0.30, USADELLAB.org, Worringerweg, Aachen, Germany) with a Phred score cut-off of 33. Data analysis was performed through the QIIME pipeline including chimera removal (version 1.9.0, Scikit-bio™, Boulder, CO, USA). Operational taxonomical unit (OTU)-picking was performed by searching the Greengenes 16S rRNA gene database [26]. Prior to analysis of the 16S amplicon sequencing data, rarefaction was used to standardize the number of sequences per sample, thus facilitating comparisons among groups. Principal Component Analysis (PCA) was performed on all of the groups based on unweighted Unifrac tables. The x- and y- coordinate values of the data points were extracted by WebPlotDigitizer [27] from the PCA plots of each colony generated in R (package FactoMineR) and were re-plotted in Microsoft Excel 2010.

### 2.4. Metabolism of GRP by Microbiome Ex Vivo

Cecal contents were diluted 1 to 25 with PBS and mixed 1:1 (*v*/*v*) with reinforced clostridial medium (RCM; BD Difco™, Franklin Lakes, NJ, USA), which was found in our earlier studies to be suitable for GRP hydrolysis ex vivo [10]. Glucoraphanin dissolved in water (final concentration 183 μM) or PBS control were added to the bacterial slurry. The mixture was incubated anaerobically at 37 °C for 120 min. Each sample was centrifuged for 1 min at 4 °C, filtered (0.22 μm), and flash-frozen in liquid nitrogen. Colon and liver tissue were also surgically removed and processed at necropsy. The first five centimeters of the colon were flushed with cold PBS, slit open lengthwise, and scraped rapidly using a histology slide on ice. The collected mucosal scrapings of the colon and known weights of the liver were snap-frozen in liquid nitrogen. All samples were stored at −80 °C until use.

### 2.5. Total ITC Quantification

Hydrolysis of GRP to ITC (SFN and erucin) by rat cecal samples was quantified using the cyclocondensation method [28], as previously described [29]. Briefly, samples were incubated with potassium phosphate buffer (25 mM) and 1,2-benzenedithiol (10 mM) for 2 h at 65 °C. After cooling to room temperature, the mixture was centrifuged at 16,000× *g* for 10 min. The supernatant was analyzed by HPLC, using a C_18_ reverse-phase column (ODS-3, 5 μm, 250 × 4.6 mm, ES Industries Marvel, West Berlin, NJ, USA) attached to a Waters HPLC system (Waters Corp., Milford, MA, USA). The solvent system was operated isocratically with 80% methanol/20% water at a flow rate of 1.0 mL/min, with 10 min column washing between sample runs. The cyclocondensation product, 1,3-benzodithiole-2-thione, was detected by absorption at 365 nm. For quantification, the peak area of 1,3-benzodithiole-2-thione (eluting between 10 and 11 min) was integrated using Empower PRO software (Waters Corp.) and compared to a standard developed by reacting known concentrations of pure SFN with 1,2-benzenedithiol.

### 2.6. NAD(P)H: Quinone Oxidoreductase (NQO1) Activity

The NQO1 activity was measured using a spectrophotometric assay as described previously [21,30] with slight modifications. For tissue preparation, rat colonic mucosa and liver were homogenized in buffer (0.05 M Tris-HCl, 1.15% KCl, 1 mM EDTA, pH 7.4) before centrifugation at 12,000× *g* for 20 min at 4 °C. Supernatants were centrifuged at 100,000× *g* for 1 h at 4 °C to separate microsomal and cytosolic fractions. The cytosolic fractions were snap-frozen in liquid nitrogen and stored at −80 °C. The cytosolic fraction was mixed (1:4 *v*/*v*) with the reaction mixture (25 mM Tris-HCl buffer, 0.67 mg/mL BSA, 0.01% Tween-20 (*v*/*v*), 0.03 mM NADP^+^, 1 mM glucose-6-phosphate, 5 μM FAD, 2 unit/mL glucose-6-phosphate dehydrogenase, and 0.72 mM 3-(4,-5-dimethylthiazo-2-yl)-2,5-diphenyltetrazolium bromide (MTT), 50 μM menadione (added immediately before reaction)). The product was measured every 50 s over 5 min at 610 nm in a μQuant plate reader (BioTek, Winooski, VT, USA). The reaction was quenched by the addition of 50 μL of 0.3 mM dicumarol in 25 mM Tris buffer (pH 7.4). The absorbance was measured continuously for another 5 min to correct for non-NQO1 specific activity (background). The protein concentrations were determined using the BioRad assay [31] with BSA as the standard. The enzyme specific activity was reported as nmol MTT reduced/min/mg protein.

### 2.7. Statistical Analysis

The nonparametric Kruskal-Wallis test was performed to compare the effect of multiple diets (CON, CB, GRP, CB-H) on the abundance of rat cecal microbial taxa (*p* < 0.05, false discovery rate < 0.1) as previously described [32]. One-tail Student’s *t*-tests were performed to compare the difference between short-term (<4 days) and long-term (>4 days) cooked broccoli feeding on rat cecal microbial communities, and to compare both the GRP hydrolysis ex vivo and the NQO1 activities between the treated and control groups.

## 3. Results 

### 3.1. Alteration in the Cecal Microbiota Community and GRP Hydrolysis Following 1–14 Days of A Cooked Broccoli Diet; Study 1

**Figure 1:**
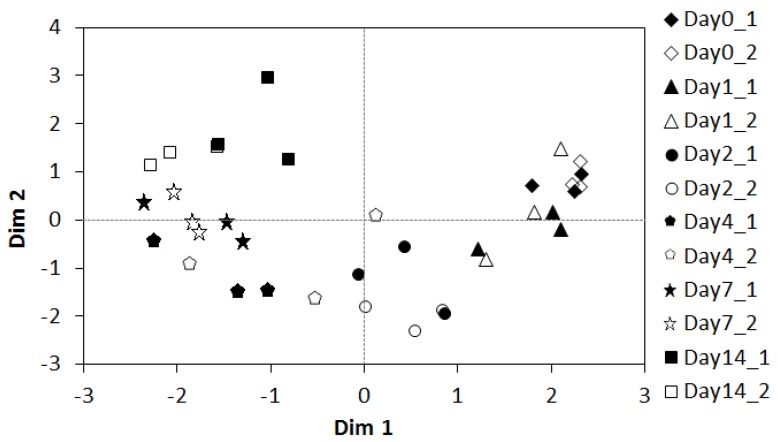
PCA-individual factor map describing the rat cecal microbial communities following 10% cooked broccoli feeding (0–14 days) from two different rat colonies. PCA analysis was performed on each colony independently and plotted on the same graph. Dim 1 = 14.47%, Dim 2 = 7.66% for rats from Colony 217 (“1”, Indianapolis IN, closed symbols) and Dim 1 = 16.48%, Dim 2 = 8.26% for rats from Colony 208 (“2”, Fredrick MD, open symbols).

The cecal microbiota was profiled to determine changes in bacterial taxa following consumption of CB or CON diets. Illumina^®^ MiSeq resulted in an average of 64,678 paired-end high-quality reads per sample. Initial PCA analysis revealed a significant shift in the cecal microbiota between two and four days on diet (Figure 1). Therefore, the cecal microbiota was reanalyzed separately for short-term (0–2 days) and long-term (4–14 days) broccoli feeding.

Metrics of alpha diversity revealed a significant increase in the number of OTUs, Chao1, Shannon, and Simpson indices in rats fed cooked broccoli for ≥4 days (Table 2).

Additional beta diversity analysis revealed six genera, mostly from the order Clostridiales (*Blautia*, *Clostridium*, *Dorea*, Ruminococcaceae (family, genus not assigned) and *Oscillospira*) significantly changed in abundance after CB feeding for four days or longer (Table 3).

**Figure 2:**
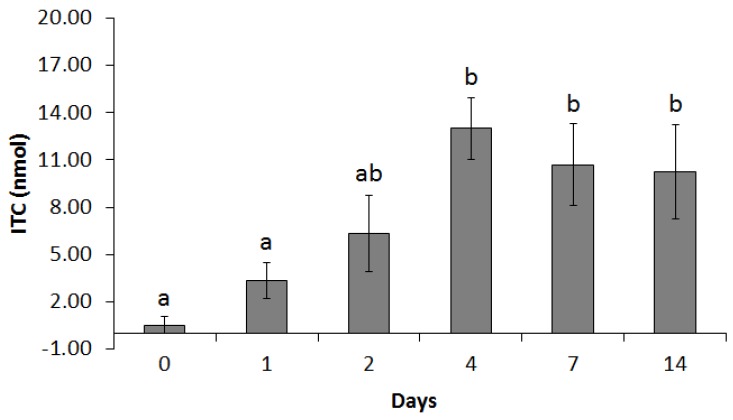
Rats (*n* = 6) were fed 10% cooked broccoli diet for 0–14 days. Concentrations of total isothiocyanate (ITC) in rat cecal supernatant after incubating ex vivo with GRP for 120 min. Values with similar letters are not different (*p* < 0.05).

In addition to the change in the microbiome, we also evaluated the myrosinase-like activity ex vivo of the cecal microbiota. Cecal samples were incubated anaerobically with excess GRP. The GRP hydrolyzing activity of the cecal microbiota increased as rats fed for longer periods, up to four days (Figure 2).

Feeding rats the broccoli diet for 4 days increased ex vivo GRP hydrolysis activity by 25-fold over that from rats fed no broccoli (CON diet) (*p* < 0.001). The enhanced GRP hydrolysis remained high throughout the feeding study (7 and 14 days). In animals receiving only the control diet, neither the microbiome community nor the GRP hydrolysis activity changed, regardless of days on the diet [33].

### 3.2. Increased NQO1 Activity in Rat Colonic Mucosa Following 1–14 Days of Cooked Broccoli Diet

**Figure 3:**
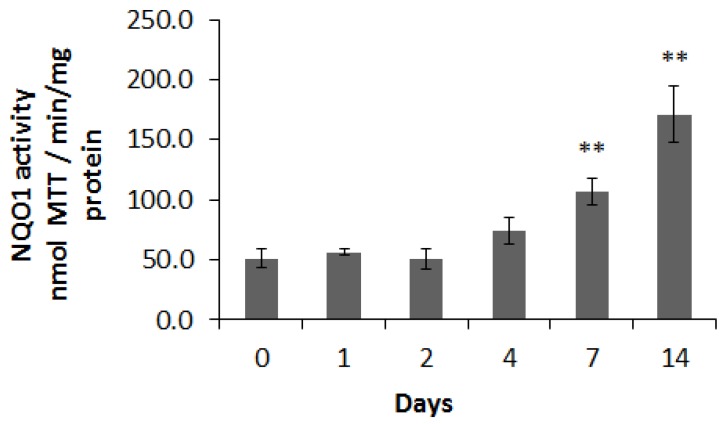
Activity of NQO1 in rat colonic mucosal tissue following cooked broccoli feeding (0–14 days; *n* = 6). Results are nmol MTT/min/mg protein. Data are mean ± SE. ** indicates significantly different from control (Day 0) (*p* < 0.01).

To assess the chemopreventive effects of CB on the host, the NQO1 activity in rat colon mucosa and liver was measured. As the rats were fed for longer periods, the colonic NQO1 activity increased gradually over 7 and 14 days (Figure 3).

The colonic NQO1 activity increased more than two-fold compared to day 0 (control diet) by day 7 (*p* = 0.004) and 3.3-fold by day 14 (*p* = 0.002). There was no increase in hepatic NQO1 activity compared to the activity in liver from rats fed the CON diet, even by 14 days of CB feeding [33].

### 3.3. Effects of GRP on Both Cecal Microbiota Composition and GRP Hydrolysis; Study 2 

Study 2 was designed to investigate whether the increased cecal microbial hydrolysis of GRP seen in Study 1 was due to the daily presence of GRP and/or non-GRP component(s) of broccoli. To this end, the composition of cecal microbiota was compared across rats fed for four days either CON, CB, GRP, or CB-H diets. On average, over 15,000 high-quality paired-end sequences per sample were obtained from Illumina^®^ MiSeq. The total numbers of OTUs and Chao1 index were greater in the CB-H group than in any other groups (*p* < 0.05; Table 4).

We performed phylogenetic beta diversity calculations to examine the dissimilarity between microbiome communities. The differences in the abundance of genera are shown as relative proportions (Table 5). Although the Shannon and Simpson indices were not different between any treatments (Table 4), phylogenetic beta diversity calculations showed that the abundance of several cecal bacterial taxa were impacted by the treatments (Table 5).

**Figure 4:**
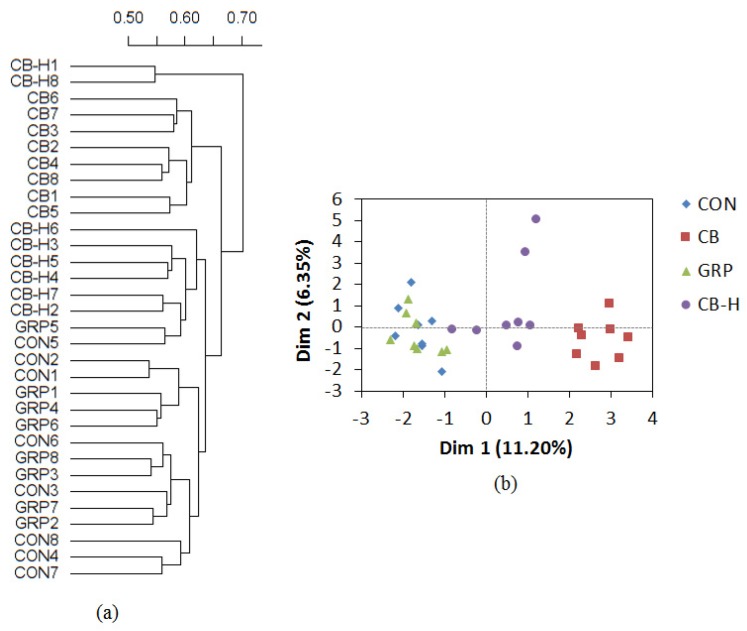
Impact of GRP and other broccoli components on rat cecal microbial communities. Rats were fed with a 10% cooked broccoli (CB), GRP-rich powder (GRP), broccoli pre-hydrolyzed using myrosinase (CB-H) or the control diet (CON) for four days (*n* = 8). (**a**) Phylogenic tree describing the clustering of cecal microbial communities; and (**b**) PCA-individual factor map describing the cecal microbial communities.

**Figure 5:**
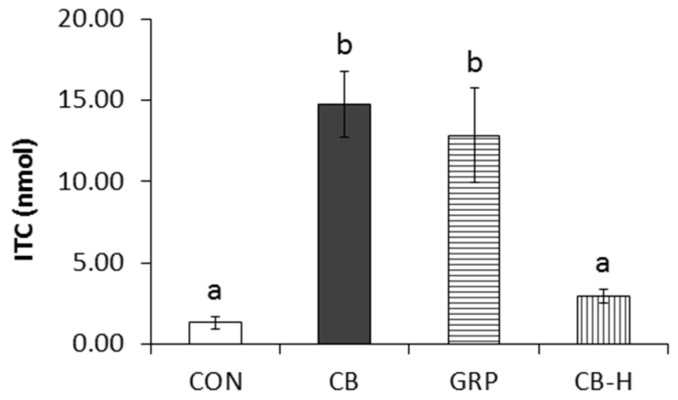
Rats were fed a 10% cooked broccoli (CB), GRP-rich powder (GRP), broccoli pre-hydrolyzed using myrosinase (CB-H) or the control diet (CON) for four days (*n* = 8). Concentrations of total isothiocyanate (ITC) were measured in rat cecal supernatant after incubation ex vivo with GRP (183 μM) for 120 min. Data are mean ± SE. Values with similar letters are not different (*p* < 0.05).

An understudied genus from the S24-7 family was found to be in greatest abundance (>15% total genera) in all groups, including from control-fed rats. The composition of the cecal microbiota from rats receiving the CB diet was quite distinct from the other groups, as observed by phylogenetic clustering (Figure 4a) and PCA analysis (Figure 4b). Conversely, in the same analysis the cecal microbiota of the CON and GRP groups were indistinguishable. Unlike those from other dietary groups, the microbiome from the CB-H group formed two unique clusters by both distance metrics. The ITC production ex vivo was similar in the CON and CB-H groups. However, production in the CB and GRP groups was greater than in either the CON or CB-H groups (Figure 5; *p* < 0.05).

### 3.4. Effect of A Raw Broccoli Diet and Its Withdrawal on GRP Hydrolytic Potential; Study 3

**Figure 6:**
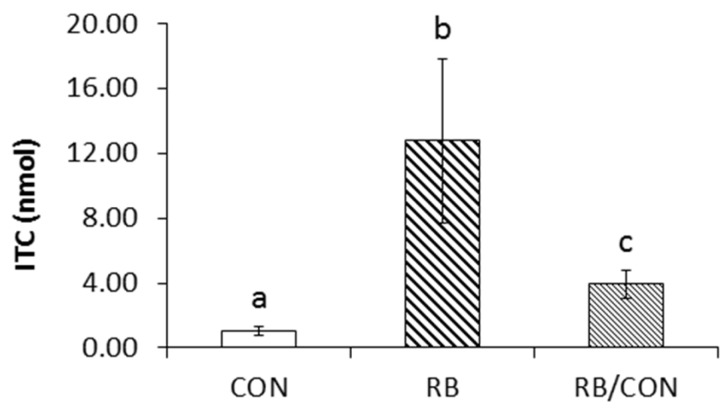
Reversibility of the impact of dietary broccoli on microbial GRP hydrolysis. Rats were fed with the control diet (CON), a 10% raw broccoli (RB) diet, or a 10% RB diet for four days, then the control diet four three days (RB/CON) (*n* = 3). Concentrations of total isothiocyanate (ITC) in rat cecal microbiota after incubation ex vivo with GRP for 120 min. Results are mean ± SE. Values with similar letters are not different (*p* < 0.05).

To determine the impact of a raw broccoli diet and its removal, we fed rats either CON diet for four days, RB diet for four days, or the RB diet for four days, followed by three days of the control diet (RB/CON). As with the cooked broccoli diet, the total ITCs produced ex vivo from cecal microbiota of rats fed raw broccoli for four days increased (*p* < 0.05) compared to ITC production by microbiota from rats on the control diet (Figure 6). However, when raw broccoli was withdrawn from the diet for three days and replaced with CON, the total ITCs production decreased, but remained greater than the CON group (*p* < 0.05).

## 4. Discussion

### 4.1. Impact of Different Diets on The Cecal Microbiota Communities

In this study, the cecal microbial communities from rats fed CB and CB-H diets were similar, yet different, from the CON-fed group (Figure 4b). Since CB contained GRP and CB-H did not, although both had a broccoli base, this indicates that non-GRP component(s) of broccoli are responsible for the new cecal microbial community structure. Further supporting the concept that GRP was not responsible for the changes seen in community structure, the cecal microbial communities from rats fed CON and GRP were similar. However, given the technical limitations of 16S rRNA sequencing, such as the short read length, the identification of bacterial changes at the species- and strain-level were not determined. Future research employing the examination of microorganisms at the species and strain level may provide further insights into the changes in the microbiome related to GRP hydrolysis.

In addition to GRP, broccoli contains several other components that may impact the microbiome [34,35]. For example, broccoli is a rich source of fiber, which can modulate the gut microbial community and improve health [36]. In the present study, fiber as a percent of the diet was maintained across all diets, although the type of fiber varied (Table 1). The flavonoids quercetin and kaempferol are also present in broccoli. These were not balanced across diets and could impact the microbiome. For example, in a study where rats were fed a high-fat high-sucrose diet, oral administration of quercetin decreased the abundance of *Erysipelotrichaceae* and *Bacillus* bacteria in the Firmicutes phylum [37].

### 4.2. GRP Hydrolysis

Early pharmacokinetic studies [13,38] reported that glucosinolate hydrolysis can occur during digestion, in the absence of myrosinase, but at a fraction of the rate of that in the presence of myrosinase. Here we show that daily broccoli feeding to rats greatly enhanced this hydrolytic rate, causing as much as a 25-fold increase in the capacity of the gut microbiota to hydrolyze GRP to bioactive SFN (Figure 2). A key finding is that it is specifically the presence of GRP in the diet that enhances microbial hydrolysis rates, since when GRP was fully hydrolyzed to SFN prior to feeding, the broccoli had no impact on rates of exogenous GRP hydrolysis (Figure 5). However at this time, we are not able to determine which bacteria perform the hydrolysis or even whether the increased rate is due to an increased number of active bacteria or to upregulation of a bacterial hydrolyzing enzyme. In a recent study [39], two bacteria isolated from human feces, *Escherichia coli* VL8 and *Enterococcus casseliflavus* CP1, were evaluated for glucosinolate metabolism. Less than 10% of the metabolized glucosinolate could be detected as ITC (6% as erucin produced, with 65% GRP loss in 8 h incubation with *Escherichia coli* VL8 and less that 1% of the starting GRP level as SFN produced vs. 53% of GRP degraded after 8 h incubation with *Enterococcus casseliflavus* CP1). It would be interesting to determine if feeding broccoli or other brassica increases the presence of these microbes or the possible reductase these authors identified. In our study, a three-day withdrawal from the broccoli diet decreased GRP hydrolysis ex vivo, suggesting that frequent broccoli ingestion may be essential for this improved microbial GRP hydrolysis. Recent studies have shown that desulfoglucosinolates can be substrates for beta hydrolase, resulting in formation of nitriles [40]. It will be interesting to see if there are bacterial N-sulfatase enzymes that are able to generate desulfoglucosinolates and whether these, by forming inactive nitriles and not reactive isothiocyanates, can protect the bacterium from glucosinolate toxicity.

In the present study, we used the cyclocondensation reaction to estimate total ITC, rather than GC or LC-MS methods that measure individual compounds, which can be summed to determine the total product formed. The cyclocondensation method is both accurate and rapid, when total metabolites are of interest, but does not provide identification of specific ITC forms. The cyclocondensantion reaction relies on the thiol compound benzene dithiol to bind to ITC and ITC metabolites. Since ITC is highly reactive, we previously proposed that ITC products of microbial hydrolysis might be bound to protein cysteines and possibly unavailable for estimation by GC or LC-MS. Using excess GSH, we were able to pull out and identify SFN and its reduced congener erucin, not seen when we performed LC-MS directly [41]. Many studies [7,10,42,43] evaluating glucosinolate metabolism by microbiota ex vivo have identified disappearance of glucosinolate substrate and/or appearance of nitriles. Since nitriles are not electrophilic, and thus not bound to protein sulfhydryls, it is possible that this is why nitriles are available for direct measurement in many studies where ITCs, such as sulforaphane, were not detected.

### 4.3. Bioactivity

Increased colonic mucosal NQO1 activity was observed after 7 and 14 days of cooked broccoli feeding. Our data clearly suggest that by frequent feeding of cooked broccoli to rats, not only the capacity of the microbiota for GRP hydrolysis was improved, but also that the ultimate NQO1 activity in rat colon was increased (Figure 3), indicating the improved potential for cancer chemopreventative activity of the host. However, no significant differences were observed in hepatic NQO1 activity [33]. In our previous study [22], we gavaged rats with different doses of GRP (30, 60, and 120 mg/kg) for four days, and observed a significant dose-dependent increase in NQO1 activity in the colon, whereas there was only a slight increase in hepatic NQO1 activity with the highest dose of GRP. The present results are consistent with our previous finding that colonic mucosa had a NQO1 response at lower doses than liver, suggesting that the dose here was sufficient to provide a local or topical effect on the gut wall, but not large enough to impact the liver.

The stimulation of colonic NQO1 activity by CB was delayed compared to the increase in GRP hydrolysis (Figure 2 and Figure 3). It would be interesting to know whether microbial hydrolysis or mucosal wall upregulation of NQO1 is the limiting step. Since GRP was essential for increased microbial hydrolysis, and because hydrolysis was increased in the microbiome from rats fed raw broccoli, this shows for the first time that some of the GRP in raw broccoli travelled to the cecum for bacterial hydrolysis rather than being completely hydrolyzed by broccoli myrosinase in the upper gut.

## 5. Conclusions

Broccoli feeding changed the composition of the rat cecal microbiota, increased glucoraphanin hydrolysis to ITC ex vivo, and increased host colonic NQO1 activity. Glucoraphanin from broccoli was responsible for the increase in the hydrolysis to ITC, whereas broccoli component(s), other than GRP, altered the cecal microbiome composition. Furthermore, withdrawal from the broccoli diet caused loss of the broccoli-enhanced GRP hydrolysis, suggesting that frequent broccoli ingestion is required to ensure optimal microbial glucosinolate hydrolysis.

## Figures and Tables

**Table 1:** Diet Compositions (%) ^1^.

Ingredients	AIN93G (CON) Diet (%)	10% Cooked Broccoli (CB) Diet (%)	10% GRP-Free Broccoli (CB-H) Diet (%)	GRP Diet (%)
Casein	20.00	17.37	17.37	19.92
Cornstarch	39.75	37.56	37.56	39.58
Maltodextrin	13.20	12.30	12.30	13.14
Sucrose	10.00	9.32	9.32	9.96
Cellulose	5.00	2.57	2.57	4.98
Mineral mix ^1^	3.50	2.69	2.69	3.49
Vitamin mix	1.00	1.00	1.00	1.00
l-cysteine	0.30	0.30	0.30	0.30
Choline bitartrate	0.25	0.25	0.25	0.25
Soybean oil	7.00	6.66	6.66	6.97
Cooked broccoli	0	10.00	0	0
Hydrolyzed broccoli	0	0	10.00	0
GRP-rich powder	0	0	0	10.00
Calculated GRP ^2^ (μmol/g diet)	0	0.26	0	0.28
Calculated SFN ^2^ (μmol/g diet)	0	0	0.17	0

^1^ The total mineral content are equal in all of the diet. ^2^ Standard deviation <0.01.

**Table 2:** Analysis of rat cecal microbiota following 0–14 days of cooked broccoli diet, study 1.

Treatment ^1^	OTUs ^2^	Chao1	Shannon	Simpson
<4 days	1393.4 ^a^ ± 84.7	3582.6 ^a^ ± 337.4	8.58 ^a^ ± 0.31	0.988 ^a^ ± 0.005
≥4 days	1481.3 ^b^ ± 105.0	3803.4 ^b^ ± 416.9	8.87 ^b^ ± 0.23	0.992 ^b^ ± 0.003
*p*-value	0.005	0.045	0.002	0.016

^1^ Microbiota from rats (*n* = 36, see Figure 1) fed cooked broccoli for fewer than four days or four days and more. ^2^ Microbial analysis by Illumina^®^ 16S rRNA sequencing (V3–V5 hypervariable region). Values within columns with the same letter are not different (*p* < 0.05). *p*-values are reported as one-tailed Student’s *t*-test. Rarefaction was calculated based on 4900 seqs per sample when the maximum number of OTUs were observed in all groups.

**Table 3:** Changes in the abundance of bacterial genera in cecal microbiome of rats (*n* = 6) fed up to 14 days of cooked broccoli diet; Study 1.

Genus	Abundance ^1^ (% Total Genera)				*p*-Value
	<4 Days		≥4 Days		
	Mean	SEM	Mean	SEM	
*Akkermansia*	0.001	0.001	**0.008**	0.003	0.007
*Blautia*	0.429	0.078	0.123	0.024	0.000
*Clostridium*	0.388	0.065	0.127	0.017	0.000
*Dorea*	1.073	0.150	0.482	0.050	0.003
*f__Ruminococcaceae; Other*	0.287	0.036	**0.497**	0.040	0.000
*Oscillospira*	9.576	0.709	**14.296**	1.171	0.003

^1^ False discovery rate < 0.1. Student *t*-test showing genera increased (bolded) and decreased (underlined) in abundance of bacterial genera from rats fed cooked broccoli for four days or more, compared to bacteria from rats fed for fewer than four days of cooked broccoli diet (*p* < 0.05). *p*-values are reported as the one-tailed Student’s *t*-test.

**Table 4:** Analysis of rat cecal microbiota following four days of treatment diets; Study 2 ^1^.

Diet	Reads Obtained from MiSeq (Min~Max)	Median Seqs per Sample after Quality Filtering (Min~Max)	% OTUs Assigned	Chao1	Observed OTUs
CON	13,242~45,673	10,917~37,747	99.88 ± 0.07	3627.1 ^a^ ± 366.6	1573.0 ^a^ ± 77.9
CB	11,516~29,303	9,447~24,029	99.89 ± 0.07	3847.5 ^a^ ± 208.8	1576.1 ^a^ ± 23.1
GRP	14,829~29,988	11,900~24,483	99.91 ± 0.05	3783.7 ^a^ ± 442.5	1557.4 ^a^ ± 122.1
CB-H	12,761~116,686	10,441~96,729	99.92 ± 0.04	3968.5 ^b^ ± 172.1	1663.6 ^b^ ± 57.4

^1^ Microbial analysis by Illumina^®^ 16S rRNA sequencing (V3–V5 hypervariable region). Rats (*n* = 32) fed four days of control (CON), cooked broccoli (CB), GRP, or GRP-free (CB-H) diet. Values within columns with the same letter are not different (*p* < 0.05). Rarefaction was performed based on 7410 sequences per sample when the maximum number of OTUs were observed in all groups.

**Table 5:** Abundance of bacterial genera (>0.1% total) of cecal contents of rats, study 2 ^1^.

Genus	Abundance ^2^ (% Total Genera)								*p*-Value
	CON		CB		GRP		CB-H		
	Mean	SEM	Mean	SEM	Mean	SEM	Mean	SEM	
*Clostridium*	0.148	0.012	**0.324**	0.055	0.185	0.027	0.109	0.017	0.007
*Coprococcus*	1.840	0.517	1.392	0.407	0.623	0.105	0.674	0.092	0.005
*Dorea*	1.229	0.150	0.357	0.046	0.772	0.095	0.598	0.117	0.000
f__Lachnospiraceae; *Other*	1.680	0.143	1.574	0.121	1.056	0.157	0.875	0.073	0.001
f__Ruminococcaceae; *Other*	1.272	0.200	1.693	0.156	0.611	0.093	1.130	0.130	0.001
f__S24-7; *g__*	15.477	1.346	13.612	1.133	15.759	1.203	**19.665**	0.706	0.007
*Prevotella*	0.119	0.049	0.163	0.083	**0.420**	0.077	**0.737**	0.285	0.003
*rc4-4*	0.965	0.165	0.214	0.035	1.063	0.159	0.592	0.081	0.000

^1^ Rat were fed four days of control (CON), cooked broccoli (CB), GRP, or GRP-removed (CB-H) diet. ^2^ False discovery rate <0.1, Student *t*-test showing genera increased (bolded) and decreased (underlined) in abundance compared to the control group (*p* < 0.05). *p*-values are reported as the Kruskal-wallis test.
